# The evolution of RHO of plant (ROP) proteins and their morphogenetic functions

**DOI:** 10.1111/nph.70455

**Published:** 2025-08-11

**Authors:** Hugh Mulvey, Liam Dolan

**Affiliations:** ^1^ National Institute for Basic Biology Okazaki 444‐8585 Japan; ^2^ Gregor Mendel Institute of Molecular Plant Biology Austrian Academy of Sciences, Vienna Biocenter Vienna 1030 Austria

**Keywords:** cell polarity, cell wall, cytoskeleton, land plants, morphogenesis, plant evolution, RHO GTPase, streptophyte algae

## Abstract

The ability of a cell to polarise, and direct cell growth or orient cell division, for example, is fundamental for the morphogenesis of multicellular organisms. A key molecular system for signalling cell polarity in diverse eukaryotes involves the RHO family of small GTPases. Since its origin in early eukaryotes, the RHO family has evolved independently in different lineages, and the plant‐specific subfamily of RHO – RHO of plants (ROP) – was established in the streptophyte algal ancestors of land plants. Insights from both bryophytes and vascular plants have revealed conserved roles for ROP signalling in land plant morphogenesis. Synthesising our current understanding of how ROP signalling evolved and how it regulates morphogenesis in extant land plants, we propose that this polarity signalling system was co‐opted to spatially coordinate morphogenetic mechanisms that evolved in the algal ancestors of land plants.

**Table:** 

	Contents	
	Summary	542
I.	Introduction	542
II.	Timeline of ROP evolution	543
III.	ROP function in land plant morphogenesis	545
IV.	ROP evolution and morphological evolution in ancestral streptophyte algae	546
	Acknowledgements	546
	References	547

## I. Introduction

RHO GTPases act as molecular switches by cycling between a plasma membrane associated GTP‐bound (active) form and a cytosolic GDP‐bound (inactive) form to regulate intracellular processes (Etienne‐Manneville & Hall, 2002). This cycling is regulated by three core interactors – guanine nucleotide exchange factors (GEFs), GTPase‐activating proteins (GAPs) and guanine nucleotide dissociation inhibitors (GDIs). These regulators determine the subcellular distribution of active and inactive RHO proteins and can themselves be influenced by RHO activity, creating dynamic feedback loops (Wu & Lew, 2013). This feedback gives rise to one of the unique properties of RHO GTPases – their ability to become locally enriched (polarised) at a specific region of the cell cortex. Polarised RHO activity promotes the asymmetric organisation of the cytoskeleton and orients membrane trafficking – both important for cellular morphogenesis. Thus, the binary nature of RHO and the dynamic interplay with its core interactors allows RHO signalling to establish intracellular asymmetry and spatially coordinate cellular morphogenesis.

Here, we review our current understanding of: (1) how a plant‐specific form of RHO – RHO of plant (ROP) – evolved; (2) how ROP influences plant morphogenesis; and (3) how the establishment of ROP signalling may have contributed to morphological evolution in the algal ancestors of land plants.

## II. Timeline of ROP evolution

### Eukaryotic origin of the RHO family of small GTPases

**Fig. 1:**
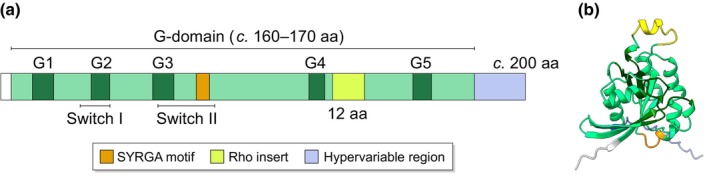
Conserved structure of RHO of plant (ROP) GTPases. (a) Schematic of domain organisation along the linear amino acid sequence of a typical ROP GTPase. Defining features of the Ras superfamily, RHO family and ROP subfamily are highlighted. All Ras superfamily members share a compact G‐domain (light green) with five conserved G‐boxes (dark green) and switch I and II regions that undergo GTP/GDP‐dependent conformational changes. The Rho insert (yellow) is found exclusively in RHO family proteins. The C‐terminal hypervariable region (light purple) present in RHO and some other Ras families is post‐translationally modified for membrane anchoring. Features, such as the SYRGA motif (orange) and the 12‐amino acid (aa) Rho insert (compared with 14‐aa typically in other RHOs), distinguish members of the ROP subfamily from those in other RHO subfamilies. (b) Tertiary structure of a typical ROP GTPase (*Marchantia polymorpha* ROP) predicted by AlphaFold. Colour coding matches that in (a). Both the Rho insert and the SYRGA motif are exposed on the protein surface.

RHO proteins, named for their homology to Ras (Ras homologous), form a family within the Ras superfamily of small GTPases (Wennerberg *et al*., 2005). The Ras superfamily members are defined by their short primary sequence (relative to other GTPases like the alpha subunit of heterotrimeric G‐proteins) encoding a conserved core G‐domain, which binds GTP/GDP and switches between the active/inactive conformations (Fig. 1). The RHO proteins additionally encode the Rho insert – a surface exposed motif – which distinguishes them from the rest of the Ras superfamily. The RHO family likely forms a monophyletic group within the Ras superfamily (Vernoud *et al*., 2003; Wennerberg *et al*., 2005; Boureux *et al*., 2007; Rojas *et al*., 2012). While Ras superfamily members have been identified in both eukaryotes and prokaryotes (Dong *et al*., 2007; Wuichet & Søgaard‐Andersen, 2015), RHO sequences have only been found in eukaryotes (Boureux *et al*., 2007; Eliáš & Klimeš, 2012). Given that RHO sequences are found in diverse eukaryotic lineages, it is likely that the RHO family originated among the Ras superfamily in the last eukaryotic common ancestor.

### ROP: a plant‐specific subfamily of RHO

Since its origin, the RHO family has evolved independently in different eukaryotic lineages, forming several distinct subfamilies. For example, in the last common ancestor (LCA) of opisthokonts – a monophyletic group that includes fungi and metazoans – at least three forms of RHO were present – Rac, Cdc42 and Rho – which each gave rise to the three major RHO subfamilies in extant opisthokonts (Boureux *et al*., 2007). Plant RHO sequences were first identified in angiosperms (Yang & Watson, 1993; Lin *et al*., 1996), and these sequences were proposed to form a plant‐specific subfamily called ROP (short for Rho‐related GTPases from plants, or RHO of plants; Li *et al*., 1998). The ROP subfamily was defined based on the observation that: (1) all the examined angiosperm RHO sequences formed a monophyletic group in a tree of eukaryotic RHO sequences; and (2) these angiosperm RHO sequences shared conserved motifs (ROP‐defining motifs) unique to them (Li *et al*., 1998; Fig. 1). All RHO sequences subsequently identified in gymnosperms, monilophytes, lycophytes and bryophytes belong to this ROP subfamily, indicating that the ROP subfamily was present in the LCA of all land plants (Fowler, 2010). Furthermore, the rescue of the *Marchantia* loss of function *rop* mutant by the *Arabidopsis ROP2* gene demonstrated that ROP function has remained conserved at least since vascular plants and bryophytes last shared a common ancestor (Rong *et al*., 2022).

### The establishment of the ROP subfamily early in streptophyte evolution

**Fig. 2:**
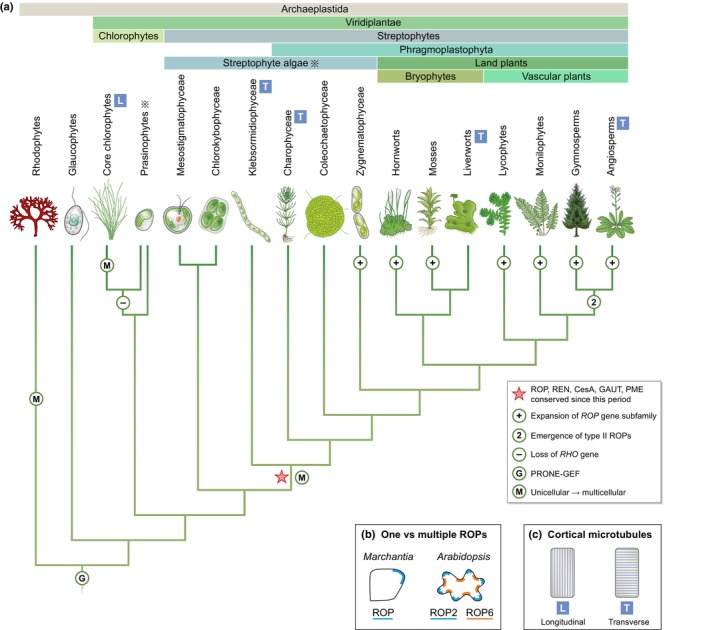
RHO and morphological evolution in plants. (a) Cladogram of major Archaeplastida lineages (topology based on Leebens‐Mack *et al*., 2019) marked with evolutionary events related to RHO GTPases and referenced in the text. The core RHO of plant (ROP) signalling module, including ROP and its regulators (e.g. ROP ENHANCER (REN)/PLECKSTRIN HOMOLOGY‐GAP (PH‐GAP)), has remained strictly conserved from the time marked with ☆. Cell wall biogenesis and remodelling enzymes, such as cellulose synthase A (CesA), galacturonosyltransferase (GAUT) and pectin methylesterase (PME), have remained conserved from the same time. The *ROP* gene subfamily expanded independently in the lineages marked with ⊕. The *ROP* gene had likely also duplicated in the common ancestor of some of these lineages (e.g. in the last common ancestor (LCA) of angiosperms and gymnosperms to give rise to type II ROPs, marked with ②); however, the precise number and timing of these earlier duplication events could not be determined with certainty. Hence ⊕ does not represent all the *ROP* gene duplication events. ⊖ denotes that the RHO GTPase encoding gene was lost sometime before the divergence of the core chlorophytes from the prasinophytes. Ⓖ PRONE‐GEF (RopGEF) is found in Viridiplantae and rhodophytes but not in taxa outside the Archaeplastida, suggesting it originated in their LCA. Ⓜ denotes that multicellularity evolved independently within the rhodophytes, core chlorophytes and streptophytes. Like streptophytes, rhodophytes and core chlorophytes also include unicellular lineages. 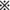 marks paraphyletic groups. (b) ROP subfamily expansion led to ROP proteins with distinct localisations and functions. (c) Transverse organisation of cortical microtubules, associated with anisotropic diffuse growth, is conserved in diverse streptophytes. Interphase cortical microtubules aligned perpendicular to the growth axis throughout the cell length have been observed in taxa marked with 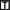 in (a).

To determine when the ROP subfamily became established during plant evolution, sequences from diverse species of Archaeplastida – the lineage comprising land plants and their algal relatives (Fig. 2a) – were analysed. This was made possible by two recent advances: first, the surge in the number and diversity of sequenced Archaeplastida genomes and transcriptomes (Leebens‐Mack *et al*., 2019); and second, improved understanding of Archaeplastida phylogeny (Bowles *et al*., 2023). Phylogenetic analyses of RHO sequences from diverse Archaeplastida species indicate that the ROP subfamily originated early in streptophyte evolution – in the LCA of land plants and the Klebsormidiophyceae (Mulvey & Dolan, 2023b). Two observations support this conclusion. First, RHO sequences from all species that descended from the LCA of land plants and the Klebsormidiophyceae constitute a monophyletic clade. Second, a ROP‐defining motif is conserved specifically in members that constitute this clade, allowing us to define this as the ROP clade. This ROP clade definition was functionally supported: the *Klebsormidium ROP* gene complemented the *Marchantia rop* mutant while the *Chlorokybus RHO* gene – from a clade sister to the ROP clade – did not (Mulvey & Dolan, 2023b). Together, the phylogenetics and cross‐species complementation experiments demonstrated that the ROP subfamily was established early in streptophyte evolution (Fig. 2a).

The establishment of the ROP subfamily early in streptophyte evolution appears to have been brought about through constrained sequence evolution rather than gene duplication. The topology of the Archaeplastida RHO phylogeny suggests a single *RHO* gene in the LCA of Viridiplantae, which was vertically inherited without duplication by the LCA of Klebsormidiophyceae and land plants, forming the ROP clade (Mulvey & Dolan, 2023b). Given that ROP regulators, such as the GAP protein ROP ENHANCER (REN)/PLECKSTRIN HOMOLOGY‐GAP, have also remained specifically conserved from this time (Fig. 2a), it is possible that the physical interaction with these ROP interactors constrained further sequence divergence, resulting in the establishment of the ROP subfamily.

### The expansion of the ROP subfamily in land plants

After the establishment of the ROP subfamily, *ROP* likely remained as a single copy gene until the emergence of the land plants. Most extant streptophyte algae encode only a single *ROP* gene, and the Zygnematophyceae ROP clade is sister to that of land plants, supporting the hypothesis that a single *ROP* gene existed in their LCA (Mulvey & Dolan, 2023b). *ROP* genes later duplicated independently in bryophytes and vascular plants (Christensen *et al*., 2003; Eklund *et al*., 2010; Fowler, 2010; Mulvey & Dolan, 2023b). While *ROP* paralogues have remained highly conserved in some lineages like mosses, they have clearly diverged in others like angiosperms. Distinct, even opposite, functions have been reported for different *Arabidopsis* ROP proteins, highlighting that ROP subfamily expansion enabled neofunctionalisation (Fig. 2b). For instance, in pavement cells of the leaf epidermis, ROP2 and ROP4 promote lobe outgrowth, while ROP6 restricts outgrowth at neck regions, to form jigsaw puzzle‐shaped cells (Fu *et al*., 2005, 2009). We propose that gene duplication in land plants relaxed the prior constraints on *ROP* sequence divergence, and consequently diversified ROP function, especially in vascular plants.

## III. ROP function in land plant morphogenesis

### Anisotropic growth

For the uniform (isotropic) turgor pressure within a plant cell to drive polarised (anisotropic) cell growth, cell wall extensibility must be anisotropic. ROP function is required for locally loosening the cell wall to promote localised growth. For example, for the tip growth of *Physcomitrium* protonema and *Arabidopsis* pollen tubes, and also for localised outgrowth of *Arabidopsis* pavement cell lobes (Fu *et al*., 2001, 2002; Cheng *et al*., 2020). This is achieved by ROP promoting the formation of fine actin filaments at the site of localised growth, which is thought to target exocytic vesicles – containing cell wall remodelling enzymes and polysaccharides – to the growth site (Lee *et al*., 2008).

ROP also promotes anisotropic diffuse growth by locally reinforcing the cell wall. In the indented neck regions of *Arabidopsis* pavement cells, ROP6 aligns cortical microtubules into parallel arrays, guiding cellulose synthase to reinforce the cell wall and restrict growth along that axis (Fu *et al*., 2009). These microtubules recruit REN, which locally inhibits ROP2 – the ROP that promotes actin accumulation and disrupts microtubule alignment – thereby reinforcing microtubule ordering through a feedback mechanism (Lauster *et al*., 2022). In *Marchantia* epidermal cells as well, ROP function is required for the parallel organisation of cortical microtubules and for anisotropic diffuse growth (Rong *et al*., 2022; Mulvey & Dolan, 2023a). It is not known whether REN also mediates this process; however, the short wavy rhizoids of the *Marchantia ren* mutant resemble wild‐type rhizoids treated with the microtubule depolymerising drug oryzalin, suggesting that the association of REN with microtubules during cellular morphogenesis is conserved within land plants (Honkanen *et al*., 2016; Champion *et al*., 2021; Sakai *et al*., 2024). Taken together, ROP function in both promoting microtubule‐mediated anisotropic diffuse growth and actin‐mediated localised growth is conserved between vascular plants and bryophytes.

### Cell division orientation

The disruption of ROP signalling in both vascular plants and bryophytes causes misoriented cell divisions, indicating its role in orienting the division plane. In some contexts, this role is linked to ROP‐mediated polarised cell growth. For example, in *Physcomitrium* subapical cell, ROP promotes polarised outgrowth and nuclear migration towards the outgrowth site, which is followed by an asymmetric cell division perpendicular to the cell growth axis (Yi & Goshima, 2020). When localised outgrowth is defective in the Pp*rop 2 3 4* triple mutant, this asymmetric cell division is defective. However, when outgrowth initiates in the mutant (due to the activity of PpROP1), the nucleus localises asymmetrically for an oblique division, suggesting that the defective positioning and orientation of the division plane is at least partly a consequence of defective polarised growth (Yi & Goshima, 2020). ROP may also regulate actin‐ or microtubule‐mediated nuclear migration independently of growth, as proposed for the asymmetric division of maize subsidiary mother cells (Humphries *et al*., 2011), although this remains to be clarified. Evidence for the most direct involvement of ROP signalling in orienting cell divisions comes from studies of its negative regulator, REN. REN interacts with kinesins at the cortical division zone (CDZ) in dividing *Arabidopsis* root cells, and its loss leads to misoriented cell divisions (Stöckle *et al*., 2016). REN localisation to the CDZ is conserved in *Physcomitrium* (Ruan *et al*., 2023) and the loss of REN function, as well as ROP overexpression, in *Marchantia* results in misoriented divisions (Mulvey & Dolan, 2023a; Sakai *et al*., 2024). This suggests that local inactivation of ROP activity by REN at the CDZ, potentially to form an actin depletion zone, is necessary to precisely orient cell divisions in both vascular plants and bryophytes (Müller, 2023).

### Cell adhesion

Plant tissue is under mechanical tension during development, requiring strong cell–cell adhesion. In both vascular plants and bryophytes, disruption to ROP signalling impairs cell adhesion (Qiu *et al*., 2002; Burkart *et al*., 2015; Bao *et al*., 2022). Since pectin in the plant cell wall is a major determinant of cell adhesion (Atakhani *et al*., 2022), it is plausible that exocytosis of pectin‐containing vesicles is defective in *rop* mutants. While reduced pectin content has not been reported in any *rop* mutants, the cell wall pectin composition influences ROP signalling. Demethylesterified pectin, which stiffens the wall, is enriched in the indented neck regions of pavement cells where it binds the receptor FERONIA to activate ROP6 signalling (Lin *et al*., 2022; Tang *et al*., 2022). This promotes the formation of parallel microtubule arrays between neck regions, reinforcing the cell wall. Therefore, as well as ROP signalling likely influencing cell wall composition through polar exocytosis, cell wall composition and mechanics can feedback to modulate ROP signalling. In *Marchantia*, the FERONIA homologue also regulates cell expansion and integrity and both Mp*fer* and Mp*rop* are defective in rhizoid tip growth and thallus development (Honkanen *et al*., 2016; Mecchia *et al*., 2022; Mulvey & Dolan, 2023a). Whether the FER‐ROP signalling mechanism, which integrates the mechanical status of the cell wall to regulate morphogenesis, is conserved in bryophytes remains to be seen.

### Independent co‐option of RHO signalling for morphogenesis in plants and opisthokonts

Although mechanisms of polarised cell growth, cell division and cell adhesion differ between land plants and the opisthokonts, RHO signalling also regulates these cellular processes in the opisthokonts. In the budding yeast, CDC42 promotes the formation of actin cables for polarised outgrowth during interphase, and Rho1 directs the assembly of the contractile actin ring during cytokinesis (Adams *et al*., 1990; Evangelista *et al*., 1997; Tolliday *et al*., 2002). For cell adhesion in animals, RHO regulation of the actin cytoskeleton is essential for forming adherens junctions comprising cadherin proteins (Hall, 1998). The mechanisms for cell growth, division and adhesion evolved separately after the opisthokont and land plant lineages diverged from their last common unicellular ancestor, suggesting that RHO signalling was independently co‐opted for these processes in the two lineages. However, the very fundamental molecular attributes of RHO, as a binary signalling switch, which can polarise and influence the cytoskeleton, have likely remained conserved since their LCA. It is tempting to speculate that these unique attributes of RHO primed it for its independent co‐option into a range of fundamental cellular morphogenetic processes.

## IV. ROP evolution and morphological evolution in ancestral streptophyte algae

Several fundamental cellular morphogenetic mechanisms that operate in land plants first evolved in streptophyte algal ancestors. As well as the phragmoplast‐based mechanism of cytokinesis, which is thought to have originated in the LCA of the Phragmoplastophyta (Fig. 2a), the mechanisms of anisotropic diffuse cell growth and cell adhesion likely evolved earlier, in the LCA of land plants and Klebsormidiophyceae.

The co‐alignment of cortical microtubules and cellulose microfibrils along the short (transverse) axis of the cell to promote anisotropic diffuse growth along the long axis has been demonstrated in the Charophyceae and land plants (Green, 1962; Paredez *et al*., 2006; Furuya *et al*., 2018). Similarly, cortical microtubules are oriented transversely in *Klebsormidium* cells during interphase (Katsaros *et al*., 2011; Fig. 2c). By contrast, cortical microtubules are predominantly oriented parallel to the long axis in filamentous chlorophyte algae, such as *Uronema* (Chlorophyceae) and *Chaetomorpha* (Ulvophyceae) (Kimura & Mizuta, 1994; Katsaros *et al*., 2011). Furthermore, in rhodophytes, cells elongate through band growth as opposed to diffuse growth, with no clear association between cortical microtubules and cell growth orientation (Waaland & Waaland, 1975; Babuka & Pueschel, 1998; Tsekos, 1999). These differences suggest that streptophytes, chlorophytes and rhodophytes – the three Archaeplastida lineages which evolved multicellularity independently – evolved distinct mechanisms of anisotropic growth. It also implies that the mechanism of aligning cortical microtubules along the short cell axis for anisotropic diffuse growth originated early in the streptophyte lineage.

At around the same time, mechanisms to maintain daughter cell adhesion likely evolved, coinciding with the emergence of multicellularity in the common ancestor of land plants and Klebsormidiophyceae. Consistently, many genes encoding cytoskeletal proteins and cell wall biogenesis and remodelling enzymes – critical for anisotropic diffuse growth and cell adhesion in land plants (e.g. cellulose synthase A, galacturonosyltransferase, pectin methylesterase) – are conserved specifically from this time (Bowles *et al*., 2020; Wang *et al*., 2020; Feng *et al*., 2024; Fig. 2a). While these streptophyte‐specific proteins carry out the physical tasks of delivering and modifying cell wall components, a mechanism was needed to spatially coordinate their activity to achieve morphogenesis.

Given its conserved role in land plant morphogenesis, and the timing of its establishment, we speculate that ROP signalling was co‐opted to spatially organise streptophyte‐specific cytoskeletal structures and direct the delivery of cell wall enzymes and components. This would have enabled precise control over cell wall anisotropy – necessary for anisotropic diffuse growth, post‐cytokinetic cell adhesion and the morphogenesis of multicellular bodies.

The co‐option of pre‐existing genes during the evolution of elaborate body plans is reported for both animals and plants (Carroll, 2008; Pires & Dolan, 2012). Rather than substantial changes in their molecular functions, changes in the spatial expression patterns of pre‐existing genes – to define distinct domains within a multicellular body – are proposed to have driven the transition from simple to more complex multicellular body plans (Carroll, 2008). In an analogous manner, gene co‐option likely formed part of the genetic basis for the unicellular–multicellular transition, which took place early in streptophyte evolution. However, rather than alterations in spatial gene expression patterns (which cannot be achieved within a single cell), changes in spatial regulation of gene products at the subcellular level – to define distinct domains within a cell – would have been needed to spatially coordinate cellular processes like cell growth, division and adhesion. The co‐option of a cell polarity signalling system, like ROP signalling, could have provided this spatial coordination.

## Competing interests

None declared.

## Author contributions

HM and LD wrote the manuscript.

## Disclaimer

The New Phytologist Foundation remains neutral with regard to jurisdictional claims in maps and in any institutional affiliations.
